# Case Report of Overlap of Diabetic Ketoacidosis and Hyperosmolar Hyperglycemic State in a 5-Year-Old with New-Onset Type 1 Diabetes Mellitus: Diagnostic and Management Considerations

**DOI:** 10.3390/reports9010027

**Published:** 2026-01-16

**Authors:** Filippos Filippatos, Georgios Themelis, Maria Dolianiti, Christina Kanaka-Gantenbein, Konstantinos Kakleas

**Affiliations:** First Department of Pediatrics, Medical School, National and Kapodistrian University of Athens, Aghia Sophia Children’s Hospital, 11527 Athens, Greecekoskakl2@yahoo.gr (K.K.)

**Keywords:** diabetic ketoacidosis, hyperglycemic hyperosmolar state, type 1 diabetes mellitus, influenza vaccination, pediatrics

## Abstract

**Background and Clinical Significance:** Overlap of diabetic ketoacidosis (DKA) and hyperosmolar hyperglycemic state (HHS) in children is a rare but life-threatening metabolic emergency. The coexistence of hyperosmolality and ketoacidosis increases neurologic vulnerability and complicates fluid and insulin management. Early identification and osmolality-guided therapy are essential to prevent cerebral edema and other complications. This case describes a 5-year-old boy with new-onset type 1 diabetes mellitus (T1D) presenting with DKA/HHS overlap two weeks after influenza vaccination—an unusual temporal association without proven causality. **Case Presentation:** A previously healthy 5-year-old presented with progressive polyuria, polydipsia, nocturnal enuresis, fatigue, and drowsiness. Two weeks earlier, he had received the influenza vaccine. Examination revealed moderate dehydration without Kussmaul respiration or altered consciousness. Laboratory evaluation showed glucose 45.9 mmol/L (826 mg/dL; reference 3.9–7.8 mmol/L), venous pH 7.29 (reference 7.35–7.45), bicarbonate 12 mmol/L (reference 22–26 mmol/L), moderate ketonuria, and measured serum osmolality 344 mOsm/kg (reference 275–295 mOsm/kg), fulfilling diagnostic criteria for DKA/HHS overlap. After an initial 20 mL/kg 0.9% NaCl bolus, fluids were adjusted to maintenance plus approximately 10% deficit using 0.45–0.75% NaCl according to sodium/osmolality trajectory. Intravenous insulin (approximately 0.03–0.05 IU/kg/h) was initiated once blood glucose no longer decreased adequately with fluids alone and had stabilized near 22.4 mmol/L (≈400 mg/dL). Dextrose was added when glucose reached 13.9 mmol/L (250 mg/dL) to avoid rapid osmolar shifts. Hourly neurological and biochemical monitoring ensured a glucose decline of 2.8–4.2 mmol/L/h (50–75 mg/dL/h) and osmolality decrease ≤3 mOsm/kg/h. The patient recovered fully without cerebral edema or neurologic sequelae. IA-2 antibody positivity with low C-peptide and markedly elevated HbA1c confirmed new-onset T1D. **Conclusions:** This case highlights the diagnostic and therapeutic challenges of pediatric DKA/HHS overlap. Osmolality-based management, conservative insulin initiation, and vigilant monitoring are crucial for preventing complications. The temporal proximity to influenza vaccination remains incidental.

## 1. Introduction and Clinical Significance

In childhood, HHS remains uncommon, but it is reported more often in youth with obesity and type 2 diabetes mellitus (T2D) than in those with type 1 diabetes mellitus (T1D) [1,2].

We present the unusual case of a previously healthy 5-year-old boy with newly diagnosed autoimmune type 1 diabetes who met criteria for overlap diabetic ketoacidosis and hyperosmolar hyperglycemic state (DKA/HHS)—a phenotype rarely reported in preschool-age children and more often described in adolescents. This report emphasizes three practical points: (i) confirmation of hypertonicity using corrected sodium and osmolality rather than glucose alone, (ii) an osmolality-guided fluid strategy with conservative correction targets, and (iii) delayed, low-dose insulin initiation once glucose decline plateaus with fluids to reduce the risk of rapid osmotic shifts. Severe hypertriglyceridemia at presentation is discussed as a marker of profound insulin deficiency and a potential source of laboratory artifacts. The temporal proximity to influenza vaccination is reported as a clinical detail; no causal relationship is inferred.

## 2. Case Presentation

The patient was a previously healthy 5-year-old boy who developed several weeks of progressive polyuria, polydipsia, nocturnal enuresis, fatigue, and reduced oral intake. Two weeks before presentation, he received the seasonal influenza vaccine; there was no history of intercurrent febrile illness, gastrointestinal symptoms, abdominal pain, recent steroid exposure, or other medications. Family history was negative for diabetes or autoimmune disease, and immunizations were otherwise up to date.

At presentation, vital signs were notable for tachycardia, age-appropriate blood pressure and respiratory rate, and normal oxygen saturation on room air. On examination in the emergency department, the child appeared dehydrated yet alert and interactive. He had dry mucous membranes and delayed capillary refill, with otherwise normal work of breathing and no Kussmaul respirations. No focal neurologic deficits were detected, and cardiopulmonary and abdominal examinations were unremarkable. Point-of-care glucose exceeded the meter range, prompting confirmation with laboratory testing.

Laboratory evaluation confirmed a mixed DKA/HHS presentation. Venous blood gas showed severe hyperglycemia (45.9 mmol/L [826 mg/dL]; reference 3.9–7.8 mmol/L), acidemia (pH 7.29; reference 7.35–7.45), and low bicarbonate (12 mmol/L; reference 22–26 mmol/L), with moderate ketonuria (50 mg/dL) consistent with DKA. Measured serum osmolality was 344 mOsm/kg (reference 275–295 mOsm/kg), and serum sodium was 129 mmol/L (reference 135–145 mmol/L; corrected 143 mmol/L), indicating marked hypertonicity and meeting the HHS criteria. Effective osmolality (calculated as 2 × Na^+^ [mmol/L] + glucose [mg/dL]/18; target fall ≤ 3 mOsm/kg/h) and corrected sodium (using +1.6 mEq/L per 100 mg/dL glucose above 100 mg/dL) were closely monitored, and therapy prioritized gradual osmolar correction to mitigate neurologic risk.

Ancillary evaluation included serial monitoring of glucose, electrolytes, and acid–base status. The trajectory of effective osmolality was closely tracked (target fall ≤ 3 mOsm/kg/h), and sodium was corrected to avoid a decline >0.5 mmol/L/h. Autoimmune testing supported new-onset T1D (IA-2 antibody positive) with low C-peptide (0.109 nmol/L; reference 0.26–0.63 nmol/L) and markedly elevated HbA1c (16%; reference < 5.7%).

Initial management followed a DKA protocol. The patient received a 20 mL/kg bolus of 0.9% NaCl over the first 30–60 min, with the option to repeat if hemodynamic instability persisted. He was started on maintenance fluids plus 5% estimated dehydration with 0.9% NaCl. Once full laboratory results confirmed marked hyperosmolality and hypertonicity, the diagnosis was refined to mixed DKA/HHS. Intravenous insulin, initially commenced at 0.07 IU/kg/h, was temporarily stopped, and fluids were adjusted to maintenance plus approximately 10% deficit using 0.45–0.75% NaCl supplemented with potassium (starting potassium concentration 40 mmol/L in the infusate), titrated to the corrected sodium and effective osmolality trajectory. This regimen continued with frequent monitoring until blood glucose, which initially fell with fluids alone, plateaued at approximately 22.4 mmol/L (403 mg/dL). At that point, intravenous insulin was reintroduced at approximately 0.03 IU/kg/h and subsequently increased to 0.05 IU/kg/h. When blood glucose reached 13.9 mmol/L (250 mg/dL), dextrose 5% was added to the intravenous fluids to prevent an excessive fall in glucose and osmolality. Electrolytes—particularly potassium, phosphate, and magnesium—were monitored frequently and supplemented according to serial measurements and urine output. Bedside glucose and effective osmolality were checked at short intervals to maintain the planned rate of correction. The patient responded well to fluid and insulin administration.

**Figure 1:**
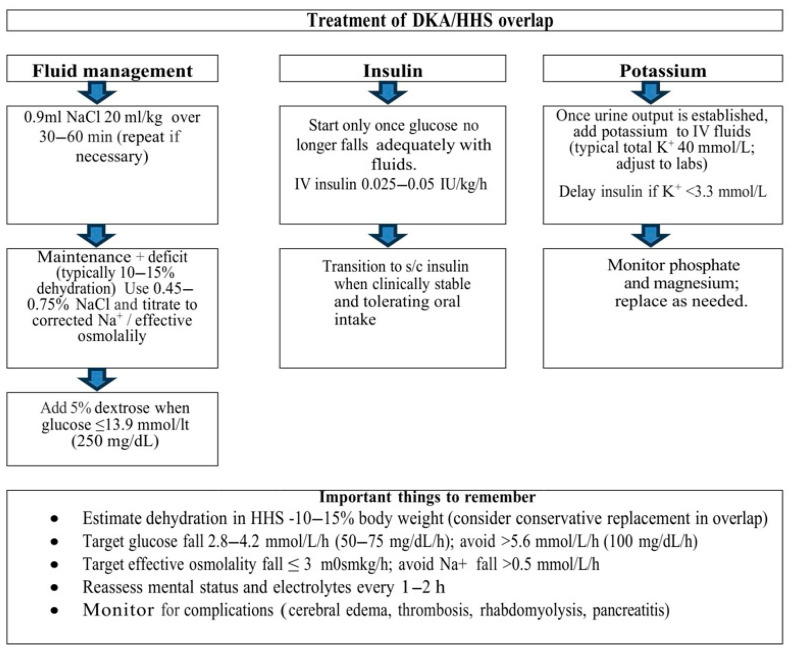
Schematic summary created by the authors outlining the most important aspects of medical treatment of mixed DKA/HHS in children, adapted from Glaser et al. [1] and Ng & Edge [3], in accordance with the ISPAD 2022 guidelines. Abbreviations: DKA, diabetic ketoacidosis; HHS, hyperosmolar hyperglycemic state; HCO_3_^−^, bicarbonate; IV, intravenous; s/c, subcutaneous; K^+^, potassium; Na^+^, sodium; NaCl, sodium chloride.

Characteristics and diagnostic criteria for DKA, HHS, and DKA/HHS overlap are summarized in Table 1. Treatment recommendations are schematically illustrated in Figure 1, and key biochemical values and treatment milestones during the initial resuscitation are summarized in Table 2.

Neurologic status remained stable without headache, irritability, or mental-status change. Ketosis resolved, and effective osmolality normalized over 24–48 h. He transitioned to a subcutaneous basal–bolus regimen, received structured diabetes education (sick-day rules and ketone monitoring), and was discharged in stable condition with a diagnosis of T1D. Early outpatient follow-up documented good clinical recovery, no recurrent decompensation, and intact neurologic examination.

## 3. Discussion

This case illustrates an uncommon but clinically significant presentation of hyperglycemic crisis in a preschool child, with overlap between DKA and HHS. It emphasizes practical points for recognition and osmolality-guided management. The child presented severe hyperglycemia, metabolic acidosis/ketonuria, and hyperosmolality, thereby fulfilling the features of both entities. Framing the evaluation around effective serum and corrected sodium was pivotal because these metrics more closely track neurologic risk than glucose alone and directly inform fluid tonicity and the timing/intensity of insulin therapy.

DKA/HHS overlap in pediatrics is under-recognized and can be missed when attention centers solely on acidosis or on absolute glucose levels. There is one previous similar case of DKA/HHS overlap reported in a 14-year-old adolescent girl in the setting of hypernatremic dehydration due to cognitive impairment and inability to express fluid intake needs [4]. There are a few other reported cases in the literature of older children with HHS following T1D, but this is the first report in a preschool child [5,6,7,8]. A structured approach—documenting pH/HCO_3_^−^, quantitative ketones, corrected Na^+^, measured/effective osmolality, and serial neurologic assessments—reduces the risk of delayed diagnosis or misclassification. In our patient, the combination of hyperglycemia, metabolic acidosis, ketonuria, and hyperosmolality established the overlap diagnosis and justified management targets that prioritized controlled osmolar correction.

A uniform consensus on the diagnostic criteria and treatment of mixed DKA/HHS remains challenging due to limited pediatric data [9]. DKA is typically defined by hyperglycemia (blood glucose > 11 mmol/L (>200 mg/dL)), a venous pH < 7.30, serum bicarbonate concentration < 18 mmol/L, and the presence of ketonemia or ketonuria, whereas HHS is characterized by blood glucose > 33.3 mmol/L (>600 mg/dL), arterial pH > 7.30 or venous pH > 7.25, serum bicarbonate concentration > 15 mmol/L, mild ketonuria, mild or absent ketonemia, and serum osmolality > 320 mOsm/kg [1]. Zahran et al. have pointed out distinct characteristics and diagnostic criteria for HHS and DKA in children and proposed a practical framework for recognizing HHS and mixed presentations [10]. Based on these criteria, our patient, with undiagnosed diabetes mellitus, blood glucose > 33.3 mmol/L (>600 mg/dL), serum osmolality > 320 mOsm/kg, and ketoacidosis, was best categorized as having DKA/HHS overlap [10].

The overlap phenotype can be conceptualized as severe insulin deficiency with concomitant hyperosmolar dehydration from prolonged osmotic diuresis [9,11]. Even modest ketogenesis, when superimposed on marked hypertonicity, amplifies neurologic vulnerability [9,11]. In addition to cerebral edema, other reported complications of HHS and mixed DKA/HHS include venous and arterial thrombosis, rhabdomyolysis, pancreatitis, and acute kidney injury, underscoring the need for meticulous monitoring of fluid balance, coagulation status, muscle enzymes, and organ function [1,12]. This dual process explains why fluid strategy, tonicity selection (0.9% vs. 0.45% NaCl), and the tempo of correction are as critical as insulin dosing. Our patient’s favorable outcome underscores the value of titrating therapy to physiology-based targets rather than fixed formulas.

The treatment of HHS is based on prompt recognition and appropriate fluid management, insulin administration, and electrolyte monitoring. In 2022, ISPAD published recommendations for the management of DKA, HHS, and mixed DKA/HHS [1]. Fluid losses in HHS usually range between 110 and 220 mL/kg, corresponding to an estimated deficit of roughly 10–15% of body weight and approximately twice that in DKA [3,13]. In our patient, we initially administered a 20 mL/kg bolus of 0.9% NaCl, followed by maintenance fluids and an estimated 10% deficit. Because of the mixed DKA/HHS presentation, we chose a conservative approach to deficit replacement, using 0.45–0.75% NaCl for ongoing maintenance and deficit correction, together with potassium supplementation (starting concentration 40 mmol/L in the infusate). Fluid management was guided by reducing effective osmolality at a rate < 3 mOsm/kg/h and by avoiding a plasma sodium decline > 0.5 mmol/L/h to minimize the risk of cerebral edema [1,14]. There is variability in guidelines regarding NaCl tonicity: some recommend 0.9% NaCl during initial resuscitation, particularly when the patient is hemodynamically unstable, followed by more hypotonic solutions (0.45–0.75% NaCl) once the circulation has stabilized and hyperosmolality persists, to avoid overcorrection and brain edema [1,3,15]. However, extensive pediatric data suggest that neither the rate of fluid administration nor the sodium chloride content alone significantly affects neurological outcome when modern protocols are followed [16].

We initially started intravenous insulin according to a DKA protocol, but this was subsequently stopped once the diagnosis of DKA/HHS overlap was established. Current pediatric guidance recommends delaying exogenous insulin administration in HHS and mixed DKA/HHS until circulation has stabilized. Fluid therapy alone no longer produces an adequate fall in blood glucose, because the combination of improved renal perfusion with glucosuria and the hypoglycemic effect of insulin can otherwise result in a rapid fall in plasma glucose, circulatory collapse, and thrombosis [1,3]. In addition, in DKA/HHS overlap, ketosis is often less pronounced than in isolated DKA, so a short delay in insulin initiation is usually not detrimental, provided careful fluid resuscitation and monitoring are in place. We restarted insulin at approximately 0.03 IU/kg/h and subsequently titrated towards 0.05 IU/kg/h, consistent with guidelines that recommend an infusion rate of about 0.025–0.05 IU/kg/h when plasma glucose levels fail to decrease by at least 2.8–4.2 mmol/L/h (50–75 mg/dL/h) with fluid administration alone [3,11].

Overall, management of mixed DKA/HHS should be individualized, integrating the child’s biochemical profile, hemodynamic status, and comorbidities, and guided by clinical judgment informed by evidence from the management of isolated DKA and isolated HHS [1,9,11].

An additional notable feature was severe hypertriglyceridemia (14.0 mmol/L [1238 mg/dL]) at presentation. Marked hypertriglyceridemia can accompany insulin deficiency through increased lipolysis and reduced lipoprotein lipase activity. From a practical standpoint, lipemia may also contribute to pseudohyponatremia when sodium is measured by indirect ion-selective electrodes, potentially underestimating true tonicity; therefore, sodium trends should be interpreted alongside measured osmolality (or direct ISE sodium) in overlap presentations. Triglycerides > 11.3 mmol/L (>1000 mg/dL) should also prompt clinical vigilance for pancreatitis and other complications, guided by symptoms and local protocols [17].

The presentation occurred two weeks after the influenza vaccination. While temporal association is factual, causality cannot be inferred from a single case, especially given weeks of antecedent polyuria/polydipsia consistent with evolving autoimmune β-cell failure. Alternative explanations (undiagnosed type 1 diabetes trajectory, intercurrent subclinical infection, variable intake) remain plausible. We therefore present vaccination timing as a clinical detail rather than a precipitating cause. This cautious framing avoids over-interpretation and aligns with best practices for case reports. Prior reports describe transient dysglycemia after influenza vaccination in some individuals with diabetes, typically resolving within 72 h; such observations do not establish a causal pathway to severe hyperglycemic crises [12]. Larger epidemiologic data would be needed to evaluate whether vaccination may, at most, act as a nonspecific stressor that unmasks pre-existing insulin deficiency rather than causing it.

Published pediatric series and case descriptions highlight that HHS and DKA/HHS overlap, although less frequently than classic DKA, and that both carry a higher risk of fluid-electrolyte derangements and neurologic complications [11]. Consistent themes include delayed recognition, underestimation of dehydration, and overly rapid osmolar shifts during treatment. Our experience reinforces three actionable lessons: (i) calculate and track effective osmolality at the bedside; (ii) defer or minimize early insulin in hyperosmolar presentations until fluids have begun to restore perfusion and dilute glucose safely; and (iii) institute standardized neurologic monitoring regardless of initial mental status.

For emergency and pediatric teams, we recommend an osmolality-first checklist in any child with glucose > 33.3–39.0 mmol/L (600–700 mg/dL): record measured and effective osmolality, corrected Na^+^, and set explicit hourly targets for glucose and osmolality decline. Quality-improvement work could test whether such checklists reduce variability in care and neurologic complications. At the research level, multicenter registries should better define the incidence, precipitating contexts, and outcomes of pediatric DKA/HHS overlap and validate physiology-based titration rules prospectively.

As a single-patient case, generalizability is constrained. We lacked immediate serum β-hydroxybutyrate quantification at presentation (qualitative ketonuria was available), and we did not perform neuroimaging because serial examinations remained normal. Nonetheless, the quantitative mapping to diagnostic thresholds, explicit therapeutic targets, and transparent reporting of calculations provide practical value.

## 4. Conclusions

This case underscores that pediatric DKA/HHS overlap can be managed safely when diagnosis is anchored to osmolality and sodium physiology and when fluids, insulin, and dextrose are titrated to conservative correction targets under close neurologic and biochemical surveillance. In a preschool child with new-onset type 1 diabetes, severe hypertriglyceridemia may be an additional clue to profound insulin deficiency and may complicate sodium interpretation; integrating measured osmolality can help prevent underestimation of hypertonicity. The temporal proximity to influenza vaccination is noted but non-causal. Clear reporting of calculations and targets may help standardize care and improve outcomes in this high-risk pediatric presentation.

## Figures and Tables

**Table 1:** Characteristics and diagnostic criteria for DKA, HHS, and DKA/HHS overlap.

Characteristic	DKA	HHS	DKA/HHS Overlap
Onset	Acute (hours–days)	Gradual (days–weeks)	Often gradual (days–weeks)
Typical population	Common in children/adolescents	More common in adolescents/adults	Uncommon
Diabetes type	Usually T1D	Usually T2D	T1D or T2D
Plasma glucose	>11 mmol/L (>200 mg/dL)	>33.3 mmol/L (>600 mg/dL)	>33.3 mmol/L (>600 mg/dL)
Effective serum osmolality	Variable	>320 mOsm/kg	>320 mOsm/kg
Ketosis	Moderate–large	Absent–small	Moderate–large
Serum bicarbonate (HCO_3_^−^)	<18 mmol/L	>18 mmol/L	<18 mmol/L
Venous/arterial pH	<7.30	Venous > 7.25 or arterial > 7.30	<7.30
Mental status	Usually alert (unless severe)	Altered mental status may occur	Varies
Key clinical features	Kussmaul breathing, abdominal pain, vomiting	Severe dehydration, tachycardia, neurologic symptoms	Mixed features

Abbreviations: DKA, diabetic ketoacidosis; HHS, hyperglycemic hyperosmolar state; T1D, type 1 diabetes; T2D, type 2 diabetes; HCO_3_^−^, bicarbonate; mOsm/kg, milliosmoles per kilogram.

**Table 2:** Key biochemical values and treatment milestones during initial resuscitation.

Time/Trigger	Key Findings/Indices	Management Decision (Rationale)
At presentation (ED)	Key findings	Glucose 45.9 mmol/L (826 mg/dL; reference 3.9–7.8 mmol/L); venous pH 7.29 (reference 7.35–7.45); HCO_3_^−^ 12 mmol/L (reference 22–26 mmol/L); ketonuria positive; measured osmolality 344 mOsm/kg (reference 275–295); Na^+^ 129 mmol/L (reference 135–145; corrected ~143); urea 6.3 mmol/L (38 mg/dL; reference 2.5–6.4); triglycerides 14.0 mmol/L (1238 mg/dL; reference < 1.7); moderate dehydration; alert.
Initial fluids	Bolus and early replacement	0.9% NaCl bolus (20 mL/kg) followed by careful rehydration with maintenance + deficit.
Early insulin	Start then pause	IV insulin 0.07 IU/kg/h started but paused due to rapid glucose fall and risk of excessive osmolar shift.
Fluids adjusted	Tonicity tailored	Switched to 0.45–0.75% NaCl (with K^+^). Goal: avoid glucose fall > 4.2 mmol/L/h (75 mg/dL/h) and effective osmolality decline >3 mOsm/kg/h.
Glucose plateau	Trigger for insulin restart	Glucose plateau (~22.4 mmol/L [403 mg/dL]) despite fluids → insulin reintroduced (0.03 IU/kg/h).
Dextrose added	Prevent hypoglycemia	Glucose 13.9 mmol/L (250 mg/dL) → 5% dextrose added; insulin maintained at 0.05 IU/kg/h.
Ongoing monitoring	Targets	Glucose decline targeted 2.8–4.2 mmol/L/h (50–75 mg/dL/h); effective osmolality decline ≤3 mOsm/kg/h; Na^+^ corrected to avoid decline >0.5 mmol/L/h. Frequent neurologic checks; no signs of cerebral edema.

Abbreviations: ED, emergency department; HCO_3_^−^, bicarbonate; IU, international units; IV, intravenous; K^+^, potassium; Na^+^, sodium; NaCl, sodium chloride.

## Data Availability

The original data presented in the study are included in the article, further inquiries can be directed to the corresponding author.
